# The Role of Nitric Oxide in Regulating Intestinal Redox Status and Intestinal Epithelial Cell Functionality

**DOI:** 10.3390/ijms20071755

**Published:** 2019-04-09

**Authors:** Kaiwen Mu, Shengwu Yu, David D. Kitts

**Affiliations:** Faculty of Land and Food Systems, The University of British Columbia, Vancouver, BC V6T 1Z4, Canada; muk@mail.ubc.ca (K.M.); tedyubca@gmail.com (S.Y.)

**Keywords:** intestinal epithelial cells, nitric oxide signaling, free radicals

## Abstract

Important functions of intestinal epithelial cells (IECs) include enabling nutrient absorption to occur passively and acting as a defense barrier against potential xenobiotic components and pathogens. A compromise to IEC function can result in the translocation of bacteria, toxins, and allergens that lead to the onset of disease. Thus, the maintenance and optimal function of IECs are critically important to ensure health. Endogenous biosynthesis of nitric oxide (NO) regulates IEC functionality both directly, through free radical activity, and indirectly through cell signaling mechanisms that impact tight junction protein expression. In this paper, we review the current knowledge on factors that regulate inducible nitric oxide synthase (iNOS) and the subsequent roles that NO has on maintaining IECs’ intestinal epithelial barrier structure, functions, and associated mechanisms of action. We also summarize important findings on the effects of bioactive dietary food components that interact with NO production and affect downstream intestinal epithelium integrity.

## 1. Introduction

Intestinal epithelial cells (IECs) exist as a continuous layer of cells that line the surface of the intestinal epithelium and display important roles in the digestion of food and the absorption of nutrients. IECs are tightly bound by intercellular junctional complexes which allow the epithelium to form a barrier that separates the luminal side from the serosal side of the cell. Specific tight junction protein mutations or aberrant signaling that disrupt the tight junction structure reduce functionality, as evidenced by the microbial invasion of interstitial tissues [1].

Nitric oxide (NO) has multiple properties that regulate IEC function. Three endogenous isoforms of NO synthase (NOS), neuronal NOS (NOS1), inducible NOS (iNOS; NOS2), and endothelial NOS (NOS3) are responsible for NO production in intestinal epithelial cells. All biosynthesis pathways use L-arginine and molecular oxygen through a complex oxygen-dependent reaction that involves electron transfer and leads to the equimolar production of NO and L-citrulline. Several co-factors, including nicotinamide adenine dinucleotide phosphate (NADPH) and tetrahydrobiopterin (BH4), are also required [2,3,4]. Nitric oxide synthase production is regulated by transcriptional and post-transcriptional mediators and cell signaling pathways.

NO has a dual role in IEC tight junction regulation. On the one hand, NO inhibits lipid and protein oxidation by directly scavenging cellular lipid or protein radicals that are formed during lipid peroxidation and protein oxidation reactions. In addition, NO has a protective role against intestinal barrier dysfunction, brought on by oxidative stress that includes hydrogen peroxide-induced changes in the protein tyrosine phosphorylation of IECs [5]. Pre-treatment of Caco-2 cells with NO has been shown to prevent a reduction in the transepithelial electrical resistance induced by H_2_O_2_ in a dose-dependent manner [5]. Tight junction proteins are closely regulated by cellular redox homeostasis. NO modulates the intestinal epithelial cell tight junction by altering the glutathione (GSH)/GSSG balance that results in the inactivation of phosphatases and protein tyrosine phosphorylation. NO also triggers a redox imbalance through a free radical mechanism that initiates lipid and protein oxidation reactions. Intracellular pathways of cell signaling are sensitive to redox changes via the modification of enzymes altered by NO. Some examples of this include the oxidation of glutathione, the inactivation of protein phosphatases directly reacting with protein and lipid radicals, and sequestering metal cofactors which deactivate enzymes. The process of S-nitrosylation or nitration of redox-sensitive proteins also alters cell signaling [6,7]. A balanced redox status is therefore paramount for the maintenance of protein structure, such as those that comprise tight junctions that enable intestinal cell functionality.

## 2. Regulation of Nitric Oxide Synthase Expression

A moderate amount of NO is continuously produced by NOS1 and NOS3, both of which are constitutively expressed in intestinal epithelial cells [8]. Most resting cells do not express iNOS, which is the primary isoform of NOS in intestinal cells, but could be induced by cytokines and some microbial products. The expression of iNOS can be constitutive or induced in vivo, for example by cytokines [9,10,11,12] during large intestinal inflammation or triggered by bacterial infections [13,14,15,16]. In IECs that are polarized, stable end-products of NO, such as nitrite and nitrate, are primarily found on the apical side. This suggests that both NO production and its reaction with metabolites are located on the apical side of intestinal epithelial cells [13]. iNOS-generated NO exerts immunomodulatory and antitumor effects through complex mechanisms in addition to possessing a number of microbial, antiviral, and anti-parasitic effects. Nonetheless, the uncontrolled induction and activation of iNOS expression is related to the pathophysiology of human diseases; therefore, iNOS expression has to be highly regulated in order to ensure overall physiological wellbeing.

The transcription of iNOS is the major regulatory scheme for NO production. Molecular iNOS transcription mechanism studies previously performed in distinct cells have indicated that different signaling pathways are triggered depending on cell types and inducers [17,18,19,20,21,22]. These in turn activate transcription factors, such as protein kinase C [23], tyrosine kinase [24], mitogen-activated protein kinases (MAP kinase) [25], and inhibitors such as protein tyrosine phosphatases [26], phosphoinositide-3-kinase [27], and nuclear factor kappa B (NF-κB) [28,29,30,31].

Recent evidence suggests that iNOS mRNA stability is highly associated with the expression of iNOS. When cells are not stimulated, even though iNOS mRNA is constitutively transcribed at basal levels, neither iNOS mRNA nor the expression of the iNOS protein can be found. These findings suggest that iNOS mRNA is probably highly unstable in non-induced cells [32,33,34]. There are a number of proteins that regulate the stability of iNOS mRNA. These proteins include protein kinase C δ (PKC) [35,36] and c-Jun N-terminal kinase (JNK) [37,38,39]. In addition, other factors that regulate iNOS mRNA stability include transforming growth factor–β (TGF-β) [40,41], dexamethasone [42,43,44], 8-bromo-cyclic guanosine monophosphate (cGMP) [45,46], intracellular calcium elevating agents [47], forskolin (activates adenylate cyclase), dibutyryl cyclic adenosine monophosphate (cAMP) (membrane permeable cAMP analog) [48,49], tetrahydrobiopterin (BH4) [50,51], and L-arginine [52,53].

The expression of iNOS is regulated by multiple signaling pathways that involve either activation [48] or inhibition [54] by cAMP-activating compounds. Conflicting evidence exists for the role of PKC and its related cytokine induction of iNOS expression, whereas both positive and negative results have been obtained with the use of PKC-activating phorbol esters or PKC inhibitors [23,55]. This may be due to the difference in the functionality of PKC in different isoforms, as discussed in several studies. Even though the level of activity of iNOS is independent of [Ca^2+^]_i_, this can also affect iNOS transcription [8,56,57]. Many studies have shown the effect of MAP kinase on iNOS expression [25,58,59,60,61].

In addition to transcriptional and post-transcriptional regulations, NO production can be modulated by a wide range of dietary components against various kinds of inducers that include oxysterols, lipopolysaccharide (LPS), tumor necrosis factor alpha (TNF-α), interleukin-1β (IL-1β), and a combination of interferon gamma (IFN-γ) and phorbol 12-myristate 13-acetate (PMA). Examples of some dietary components with this bioactivity include a number of polyphenolic compounds naturally present in fruits and vegetables [62,63,64,65,66], bioactive peptides derived from milk proteins [67], amino acids [68], or products of the Maillard reaction [69] (Table 1). Ingested nitrate is taken up in salivary glands which secrete it for further transformation by facultative anaerobic bacteria to nitrite—an important example of biological symbiosis where humans do not possess the required nitrate reductases. Nitrite is further reduced to NO as a result of low stomach pH, which represents another source of inorganic nitrate; this is particularly important as it represents a source of NO for the regulation of gastric mucosal blood flow.

## 3. NO Regulation of Intestinal Tight Junction Function

**Figure 1:**
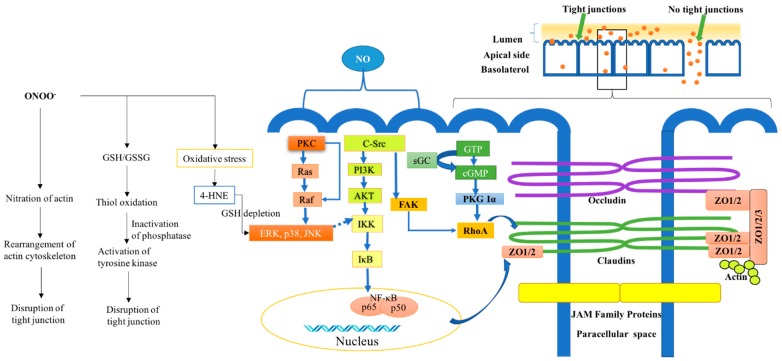
Involvement of NO in cellular signaling activity.

IECs are vital components for the paracellular absorption of nutrients [73]. In recent years, a number of tight junction proteins have been discovered, and some have been shown to function as scaffolds that link integral proteins with the actin cytoskeleton (Figure 1) [73]. Some tight junction proteins function to cross-link transmembrane junction proteins [73], while some are involved in cell signaling through an association with kinases and Ras. There are some sub-membranous junctional proteins which also contribute to gene expression because of specific binding properties to transcription factors. Occludin and claudin proteins, for example, are common integral proteins that constitute the backbone of tight junction strands. Some studies have emphasized the role of iNOS in tight junction disruption. NO, for example, can be an important mediator in the regulation of intestinal permeability [74]. However, the over-production of NO synthesized by iNOS can be attributed to intestinal barrier dysfunction [9]. A low level of NO synthesized by constitutional nitric oxide synthase appears to be a homeostatic control to optimize its function. On the other hand, similar to the intracellular synthesis of NO by iNOS, it is noteworthy that NO is also produced extracellularly by a NO donor, such as sodium nitroprusside or S-nitroso-N-acetylpenicillamine (SNAP); both of which have a detrimental effect on tight junction function [9]. Tang et al. [75] used in vitro monolayer cell-based and in vivo mouse studies to show that alcohol induced the dysregulation of intestinal tight junction proteins, such as zonula occludens-1 (ZO-1). This was ascribed to the upregulation of intestinal iNOS by alcohol. In other words, iNOS signaling has an indispensable role in alcohol-induced ZO-1 disruption [75]. Studies performed by Han and others support that NO plays a role in compromised epithelial barrier function. They showed that cytokines decrease epithelial barrier function by decreasing the expression of ZO-1, ZO-3, and occludin protein; moreover, these effects could be modulated by the NO scavenger 2-(4-carboxyphenyl)-4,4,5,5-tetramethylimidazoline-1-oxyl-3-oxide (cPTIO) [74]. On the other hand, NO may protect tight junctions against reactive oxygen species-induced dysfunction. Studies have shown that hydrogen peroxide-induced tight junction protein phosphorylation and further barrier disruption was mitigated by adding a NO donor [5].

## 4. Effect of Nitric Oxide on Intestinal Epithelial Tight Junctions: Mechanism of Action

### 4.1. The Involvement of Nitric Oxide in Cellular Signaling Activity

In general terms, the known mechanism of action of NO on redox balance includes a direct effect, where NO directly reacts with target molecules, i.e., metal centers and lipid radicals. Alternatively, NO has an indirect effect on redox, where it first reacts with reactive oxygen species (ROS), such as O_2_ or O_2_^•−^ to form ONOO^−^ or ONOOH [76,77], prior to reacting with the target molecule. The mechanism behind nitric oxide overproduction-induced abnormal intestinal barrier function includes the effect of NO on redox balance and on cell signaling, which further influences tight junction function [78,79].

#### 4.1.1. Binding to Metal Centers

A major target of NO-regulated cell signaling is soluble guanylycylase (sGC), a heme-containing enzyme that converts GTP to cGMP, an important signaling molecule. NO activates sGC by binding to its heme group, leading to changes in the porphyrin ring structure. sGC is activated in the presence of low NO concentrations (10–100 nM), and the activation of sGC leads to a significant increase in the rate of cGMP synthesis, which in turn activates protein kinase (PKG) upon the binding of cGMP [80]. Cyclic guanosine monophosphate-dependent PKG Iα binds directly to and also regulates a Ras homolog gene family, member A (RhoA) [81]. RhoA is a small G-protein in the Rho family. Studies have shown that it can regulate tight junction protein expression by modulating Claudin-2 expression [82] and actin [83,84], as shown in Figure 1.

#### 4.1.2. S-Nitrosylation on Redox Sensitive Cysteines of Susceptible Proteins

NO reacts directly with thiol-containing amino acids and induces the formation of S-nitroso-thiols from cysteine residues (termed S-nitrosylation). The reaction results in the altered function or deactivation of enzymes [8,85]. MAPK signaling has an important role in cellular activities including regulating tight junction protein expression [86]. Three well-characterized MAPK families identified are extracellular signal-regulated kinase (ERK), Jun kinase (JNK/SAPK), and p38 MAPK. NO is implicated in MAPK signaling by nitrosylating cysteine-118 in p21^Ras^, a kinase involved in the MAP kinase cascade activation [87]. Another example is the S-nitrosylation of c-Src, which further activates the NF-κB pathway. The activation of c-Src also affects RhoA by activating focal adhesion kinase (FAK). S-nitrosylation may also occur with the NF-κB subunit p50 (residue Cys 62). These signaling activities involving a redox-sensitive nuclear transcription factor (NF-κB) are critical for the regulation of tight junction proteins [88,89,90,91,92,93]. In quiescent conditions, NF-κB is bound to a family of inhibitory proteins known as NF-κB inhibitors (I-κBs), and the complexes are retained in the cytoplasm [94]. The phosphorylation of I-κB by the the I-κB kinase (IKK) complex activates the NF-κB, causing I-κB degradation by the ubiquitin-proteasome pathway (UPP). The released NF-κB translocates to the nucleus and binds to promoters on target genes, thereby regulating tight junction protein expression [88]. In addition, there is feedback on NF-κB, a known mediator of iNOS expression which in turn regulates NO production. Studies have shown that NO also affects NF-κB activity by inhibiting the DNA binding of recombinant NF-κB p50 and p65 homodimers as well as p50/p65 heterodimers [95]. The inhibition of p50 DNA binding by NO production can lead to modification of the conserved redox-sensitive C62 residue [95].

Signaling pathways are sensitive to the redox environment; hence, NO affects signaling pathways due to its involvement in pathways that alter redox status. For example, the Keap1/Nrf2-ARE pathway has an important role in protecting cells from oxidative stress [96]. Under normal conditions, the transcription factor Nrf2 is bound to the actin-anchored protein Keap1, and this interaction retains the Nrf2 in the cytoplasm while maintaining a low basal expression of Nrf2-regulated genes [96]. Human Keap1 has 27 cysteine residues, and several are known to be primary sensors for stress signals. Hence, modification on these critical cysteine residues produces conformational changes in Keap1, which liberates Nrf2 from Keap1. After being released from Keap1, Nrf2 translocates to the nucleus and transactivates the expression of cytoprotective genes, thereby enhancing cell survival. NO may contribute to the nuclear accumulation of Nrf2 by S-nitrosylation of Keap1, thus facilitating its dissociation from Keap1 [96]. The activation of Nrf2 upregulates antioxidant response enzymes that lead to the maintenance of optimum intracellular glutathione levels to ensure a redox balance is obtained for peak tight junction protein functionality.

#### 4.1.3. Reactions of NO with Proteins that Affect Cell Signaling

Peroxynitrite leads to protein nitration. Protein tyrosine nitration refers to a covalent protein modification process that occurs from the addition of a nitro (-NO_2_) group onto the *ortho* carbons on the aromatic ring of tyrosine residues, catalyzed primarily by metalloproteins [6]. Nitrogen dioxide has been found to be capable of nitrating tyrosine residues [97,98,99].

NO also reacts with thiol groups as evidenced by the relevant example involving the glutathione (GSH/GSSG) redox system, a critical balance for cell survival. Changes in the GSH/GSSG ratio buffer reflect changes in intracellular redox alterations [100]. Under physiological conditions, the reduced form of glutathione is 10- to 100-fold higher than its oxidized form. Studies have shown that changes in this ratio may modulate several cellular reactions involved in signal transduction [101,102]. When cells are subjected to oxidative stimuli, the GSH/GSSG ratio tends to decrease, by either increasing the amount of oxidized glutathione or decreasing the amount of reduced glutathione. The oxidation of the thiol group of glutathione leads to the inactivation of phosphatases, which further activates tyrosine kinases. The activation of tyrosine kinase leads to the disruption of the tight junction protein [103].

NO has a critical role in modulating glutathione homeostasis; by oxidizing GSH and generating GSNO, transnitrosylation reactions are mediated [104]. In addition, the ONOO^−^ produced from NO reacting with O_2_^•−^ interact with reduced glutathione and lead to the reversible S-glutathionylation of proteins [104].

In summary, NO has been shown to mediate post-translational modifications of proteins, representing a mechanism for cell signaling. The major four modification pathways are: (i) binding to metal centers, (ii) NO-induced formation of S-nitrosylation on redox-sensitive cysteines of susceptible proteins, (iii) nitration of amino acids including tyrosine and tryptophan, and (iv) oxidation of thiols, including cysteine and methionine residues and tyrosine [6,7]. Some examples of the effect of NO on cell signaling are discussed herein. In addition, NO can interrupt protein oxidation via inhibiting the formation of radicals produced in lipid and protein oxidation reactions.

### 4.2. Effect of NO on Protein Oxidation and Interference with Lipid and Protein Oxidation Reactions

**Figure 2:**
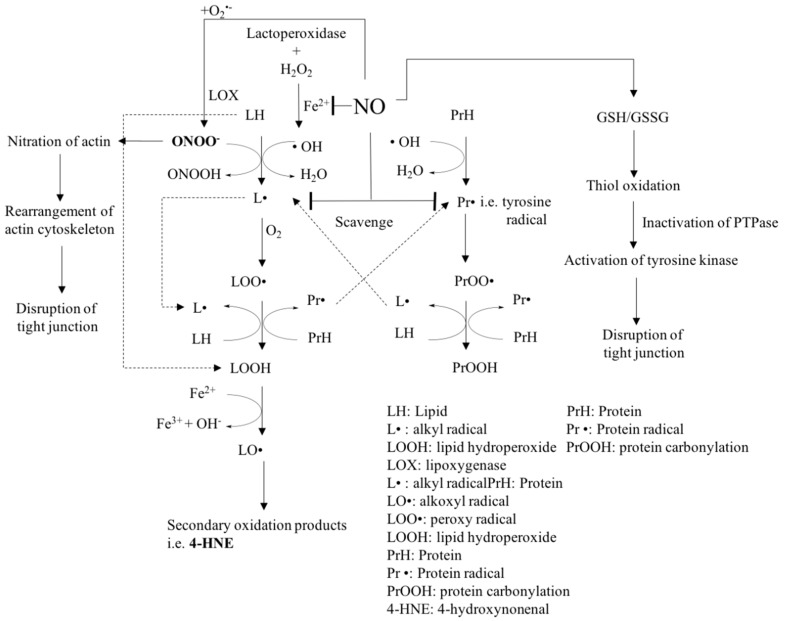
Examples of mechanisms that are involved with nitric oxide on epithelial barrier disruption by modulating the redox homeostasis of cells [103].

The oxidation of membrane lipids occurs with unsaturated fatty acids that are susceptible to both enzymatic and non-enzymatic oxidation mechanisms (Figure 2). Enzymatic reactions of lipids proceed in the presence of lipoxygenases (LOX), a family of iron-containing enzymes that transform polyunsaturated fatty acids (PUFA) to lipid hydroperoxides (LOOH) [105]. The non-enzymatic oxidation of lipids is initiated by a free radical chain reaction that results in the formation of peroxyl radicals [106,107], which in turn oxidizes other molecules, thereby inducing a chain reaction. Figure 2 shows the sequence of reactions involved in the lipid oxidation scheme.

Free radical chain reactions are initiated and form hydroxyl radicals; the formation of hydroxyl radicals is accelerated when in the presence of transition metal compounds, such as iron [108]. This leads to the abstraction of hydrogen from an unsaturated fatty acid to produce a fatty acid radical termed alkyl radical (L**·**). Thereafter, the addition of oxygen to the L**·** contributes to the formation of a peroxyl radical (LOO**·**). Due to its high energy level, the abstraction of another hydrogen from the unsaturated lipid occurs, which leads to the formation of a lipid hydroperoxide (LOOH) and a new L**·** on another unsaturated fatty acid molecule. Lipid hydroperoxides are referred to as primary oxidation products, which are further reacted in a Fenton reaction that yields alkoxyl radicals (LO**·**). β-scission reactions occur which yield secondary lipid oxidation products, such as aldehyde malonaldehyde [106,108]. Similar to lipid oxidation, protein oxidation pathways follow parallel free radical chain reactions [109,110,111]. Lipid hydroperoxides can lead to the formation of protein-centered radicals [112]. Oxygen can be added to protein-centered radicals with the formation of a protein peroxyl radical (PrOO**·**), and the high energy of PrOO**·** abstracts hydrogen from another protein molecule, thereby leading to the formation of protein carbonyls (PrOOH) [111]. In the presence of peroxidizing lipids, proteins form protein–protein cross-linked structures which result in the reduction of protein solubility [112]. The protein–protein cross-linkages proceed via the collision of two protein radicals that result in a termination reaction [113]. In addition to protein–protein cross-linkages, lipid–protein cross-linkages also occur [112]. Protein scission and amino acid damage may also occur [112].

Amino acid side chains are susceptible to free radical attack, depending on the type of amino acid. For example, cysteine is the most susceptible amino acid residue, and it is often preferentially oxidized [114]. In particular, amino acids which have a sulfhydryl amino group, thioether, indole ring, and imidazole rings, respectively, as side groups are relatively more sensitive to oxidation reactions initiated by oxidizing lipids and related products [112,115]. Thus, cysteine, methionine, lysine, histidine, arginine, as well as tryptophan residues are common targets of free radicals generated via lipid oxidation [114].

Secondary oxidation products, such as malondialdehyde (MDA) and 4-hydroxynonenal (4 HNE), react with proteins by forming covalent linkages [112] and lead to intra- or inter-molecular cross-linking [116]. These conformational changes that result in the formation of protein adducts lead to the aggregation or precipitation of proteins, thereby affecting normal protein degradation pathways [117]. It is important to point out that lipid peroxidation products such as 4-HNE can also influence tight junction stability by modulating glutathione levels and the MAPK signaling pathway.

NO also acts as an antioxidant to scavenge peroxyl radicals and therefore is involved in inhibiting lipid peroxidation propagation reactions [118,119,120,121,122,123]. NO is not a strong oxidant per se and therefore is unable to extract bis-allylic hydrogen from PUFA, which is needed to initiate the peroxidation chain reaction [124]. In addition to non-enzymatic lipid oxidation, NO affects enzymatic lipid oxidation by inactivating enzymes, such as cytochrome p450, lipoxygenases, or cyclooxygenases [125], through a reduction of the active site non-heme iron or heme to an inactive ferrous form.

NO also has indirect effects mediated by reactive nitrogen species (RNS) that are produced by the interaction of NO with superoxide anions, yielding the formation of highly reactive peroxynitrite (ONOO^-^). The highly reactive peroxynitrite (ONOO^-^) initiates lipid peroxidation in a radical manner [124,126,127,128]. At the same time, the RNS (ONOO^-^) is less reactive compared to the superoxide radical.

Among these mechanisms of action, the reaction route for NO within a cell is determined by three main criteria that include the degree of exposure, the availability of target molecules, and the structure of target proteins [129]. Flux rates of NO and its sources determine the degree of NOS exposure to NO. For example, the reaction of NO with superoxide, molecular oxygen, or thiol groups is dependent on the flux rate of NO relative to the concentration of these target molecules [129].

## 5. Conclusions

NO has multiple effects on intestinal epithelial cell functionality that involve numerous complex intracellular and molecular mechanisms of actions. A balanced redox status is a central and critical factor for the maintenance of pathways important for IEC functionality. Thus, modulating NO to maintain cell redox homeostasis is one important underlying mechanism to ensure optimal intestinal tight junction function. This is a prerequisite for a healthy gut despite the potential for frequent exposure to potential toxins (allergens) and pathogens that can be consumed simultaneously or at different times from the diet.

## Figures and Tables

**Table 1:** Effect of dietary component on nitric oxide production in intestinal epithelial cells.

Dietary Component	Inducer	Cell Type	Description	Ref
Olive oil polyphenols	Oxysterols	Caco-2 cells	Oxysterols induced nitric oxide (NO) generation was suppressed by tested compounds	[70]
Hydroxytyrosol and tyrosol metabolites	Lipopolysaccharide (LPS)	Caco-2 cells	LPS-induced NO release was inhibited by tested compounds	[71]
Cinnamon	LPS	Caco-2 and Raw 264.7 co-culture	LPS-induced NO release was inhibited by tested compound	[72]
Gastrointestinal-digested blackcurrant extracts	LPS	Caco-2 and Raw 264.7 co-culture	LPS-induced NO release was inhibited by tested compounds	[63]
Purple carrot anthocyanins	LPS	Caco-2 and Raw 264.7 co-culture	LPS-induced NO release was inhibited by tested compounds	[64]
Bovine and soybean milk bioactive compounds	LPS	Caco-2 cells	LPS-induced NO release was inhibited by tested compounds	[67]
Resveratrol	LPS	Caco-2 cells or SW480	LPS-induced NO release was inhibited by tested compound	[65]
Lutein	Tumor necrosis factor (TNF)-α	Caco-2 cells	TNF-α was suppressed by tested compound	[66]
L-arginine	Interleukin (IL)-1β	Caco-2 cells	IL-1β-induced NO release was inhibited by tested compound	[68]
Maillard reaction products	IFN-γ + phorbol 12-myristate 13-acetate (PMA)	Caco-2 cells	Induced NO release was inhibited by tested compounds	[69]
